# Elderly Fall Detection Based on GCN-LSTM Multi-Task Learning Using Nursing Aids Integrated with Multi-Array Flexible Tactile Sensors

**DOI:** 10.3390/bios13090862

**Published:** 2023-08-31

**Authors:** Tong Li, Yuhang Yan, Minghui Yin, Jing An, Gang Chen, Yifan Wang, Chunxiu Liu, Ning Xue

**Affiliations:** 1 School of Modern Post (School of Automation), Beijing University of Posts and Telecommunications, Beijing 100876, China; yanyuhang@bupt.edu.cn (Y.Y.); jingan@bupt.edu.cn (J.A.); chengang_zdh@bupt.edu.cn (G.C.); 2 State Key Laboratory of Transducer Technology, Aerospace Information Research Institute, Chinese Academy of Sciences, Beijing 100190, China; minghuiyin23@163.com (M.Y.); cxliu@mail.ie.ac.cn (C.L.); xuening@aircas.ac.cn (N.X.); 3 School of Electronic, Electrical, and Communication Engineering, University of Chinese Academy of Sciences (UCAS), Beijing 100190, China; 4 School of Artificial Intelligence, Beijing University of Posts and Telecommunications, Beijing 100876, China; wangyifan@bupt.edu.cn

**Keywords:** elderly fall detection, multi-array flexible tactile sensor, nursing aids, tactile sequences, multi-task learning

## Abstract

Due to the frailty of elderly individuals’ physical condition, falling can lead to severe bodily injuries. Effective fall detection can significantly reduce the occurrence of such incidents. However, current fall detection methods heavily rely on visual and multi-sensor devices, which incur higher costs and complex wearable designs, limiting their wide-ranging applicability. In this paper, we propose a fall detection method based on nursing aids integrated with multi-array flexible tactile sensors. We design a kind of multi-array capacitive tactile sensor and arrange the distribution of tactile sensors on the foot based on plantar force analysis and measure tactile sequences from the sole of the foot to develop a dataset. Then we construct a fall detection model based on a graph convolution neural network and long-short term memory network (GCN-LSTM), where the GCN module and LSTM module separately extract spatial and temporal features from the tactile sequences, achieving detection on tactile data of foot and walking states for specific time series in the future. Experiments are carried out with the fall detection model, the Mean Squared Error (MSE) of the predicted tactile data of the foot at the next time step is 0.0716, with the fall detection accuracy of 96.36%. What is more, the model can achieve fall detection on 5-time steps with 0.2-s intervals in the future with high confidence results. It exhibits outstanding performance, surpassing other baseline algorithms. Besides, we conduct experiments on different ground types and ground morphologies for fall detection, and the model showcases robust generalization capabilities.

## 1. Introduction

The world’s population is gradually moving towards aging [1,2,3]. According to the “2023 World Social Report”, as of 2021, the global population aged 65 and above has reached 761 million. It is projected to increase to 1.6 billion by 2050, representing a staggering 110.2% growth. Along with the demographic shift comes the escalating issue of elderly health concerns. The incidence of falls among the elderly has become a critical global challenge in this new era. Approximately 32% of elderly individuals report experiencing falls each year, with 20% of them requiring medical attention, and one-tenth of the cases leading to fractures [4,5,6]. In the United States, there is an elderly emergency every 15 s, and an elderly person dies from a fall every 29 min [7,8,9]. Concurrently, the public health system costs for elderly individuals injured due to falls are escalating worldwide, imposing significant direct or indirect expenses on families, communities, and society [10,11,12]. Preventing falls among the elderly [13,14,15] can reduce the need for medical care resulting from fall-related injuries, alleviate social costs and medical burdens, and help elderly individuals avoid issues like restricted mobility, self-confidence diminishment, and social isolation caused by falls. Despite these challenges, research on elderly fall detection remains relatively insufficient. Therefore, this study aims to propose a fall detection method to enhance safety during the mobility of elderly individuals.

The occurrence of falls during the mobility of elderly individuals varies significantly, and different fall directions can provide further insights into specific joint vulnerabilities and potential fractures. Luukinen et al. [16] have summarized the correlation between different fall patterns and fracture occurrences. A majority of elderly falls indeed happen forward, this may be attributed to factors such as an unstable gait, diminished balance, or weakened visual perception. However, sideways falls equally pose a serious threat to the elderly. Sideways falls can occur during walking, for instance, stepping on uneven ground or tripping over obstacles. Furthermore, osteoporosis and reduced muscle strength in the elderly increase the risk of sideways falls. Hence, accurately determining and distinguishing the direction of falls holds crucial significance in preventing and intervening elderly falls. Chia [17] classifies fall types based on observed characteristics into forward, backward, left, or right, while Mustafa et al. [18] adopt four fundamental fall types (forward, backward, left, and right) and validate the effectiveness of this classification method in covering the majority of fall scenarios. Our primary objective is to detect and identify the direction of falls. Based on the aforementioned research, we categorize elderly locomotion into two states: normal and fall, with falls further divided into four directions: forward, backward, left, and right.

Existing gait detection algorithms primarily rely on visual devices and multi-sensor equipment. Visual devices employ deep learning and image processing techniques to detect falls by analyzing images or video data. For instance, Saturnino et al. [19] achieved fall detection for single or multiple individuals using RGB cameras via fall classification algorithms. Han et al. [20] extracted motion feature flows to identify potential fall frames and used lightweight classification networks on mobile devices for fall classification. However, these algorithms demand significant computational resources, making real-time and rapid fall detection during elderly individuals’ mobility challenging. Additionally, visual detection methods are susceptible to obstructions in situations with limited device placement, which may hinder timely fall detection and increase the risk of accidents. Considering the limitations of visual devices, some researchers have employed devices integrated with multiple sensors, such as accelerometers, gyroscopes, pressure plates, or radio sensors, to monitor body posture, gait parameters, ground reaction forces, and other metrics. By fusing and analyzing data from multiple sensors, accurate fall detection can be achieved. Bruno et al. [21] classified falls and daily activities by fusing signals from smartphone gyroscopes and accelerometers. Georgia et al. [22] analyzed and predicted leg positions and corresponding gait phases by fusing data from multi-modal RGB-D and non-wearable sensors like Laser Range Finder (LRF). However, multi-sensor devices also come with certain drawbacks. On the one hand, they require more hardware components and technical integration, leading to increased system complexity. Moreover, complex wearable sensors may not be user-friendly for elderly individuals with limited mobility. On the other hand, multi-sensor devices typically necessitate additional energy supply and computational resources for data collection, processing and analysis, which may limit their usage time and hinder long-term fall warning capabilities.

In recent years, tactile sensors have witnessed extensive development and application. These sensors are designed to measure contact force or pressure and possess highly sensitive characteristics, capable of detecting even minute changes in contact force and pressure. Additionally, flexible tactile sensors exhibit remarkable flexibility and adaptability to irregular shapes and surfaces. In fall detection, tactile sensors can be employed to monitor the contact situation and pressure distribution between the human foot and the supporting surface. By analyzing tactile data, fall events can be identified. Duclos et al. [23] validated the effectiveness of the stable/unstable force model in assessing human dynamic balance by using infrared markers and pressure plates. Liu et al. [24] integrated data from four single-point tactile sensors placed under shoe insoles with data from a three-axis accelerometer at the waist and achieved fall prediction for elderly individuals through a BP neural network algorithm. Wei et al. [25] utilized an acceleration sensor and a single-point tactile sensor detection module to collect data on user acceleration and hand-touch force, extracting features and conducting fall detection through time-domain feature representation and recognition algorithms. Wang et al. [26] proposed a multi-sensor fusion fall detection method that combines tactile and other sensors to effectively distinguish fall events from daily activities. The approaches mentioned above demonstrate the immense potential of tactile sensors in fall prediction. Existing fall prediction methods based on tactile sensors are primarily implemented by installing sensors fixedly on the ground or at corresponding positions on the human body. However, the former approach is limited by layout constraints, which may result in incomplete coverage or measurement of pressure distribution in specific areas, leading to insufficient information. The latter approach often relies on single-point sensors, which have limited representational capabilities and may not fully reflect the real contact status, necessitating the combination of data from multiple sensors for effective detection. However, existing methods can only detect the current walking state and are unable to predict the walking states for multiple time steps.

To address the issues of multi-sensor layout, limited performance, and weak tactile perception representation, this paper proposes an accurate and efficient elderly fall detection method using a multi-array tactile sensor integrated with nursing aids. The method analyzes the foot structure of elderly individuals and designs a layout for tactile sensors, which are installed on the soles of the nursing aids. By analyzing the tactile data collected during the walking process, a tactile time-series dataset for fall detection is constructed. Based on this dataset, the walking states are categorized into five classes: forward, backward, leftward, rightward tilts, and normal walking. A multi-task learning GCN-LSTM model of fall detection is built, capable of predicting the future states of walking over multiple time steps, thereby achieving effective early warnings and ensuring the safety of elderly individuals during their mobility.

## 2. Materials and Methods

### 2.1. The Design and Performance of the Tactile Sensor Array

**Figure 1:**
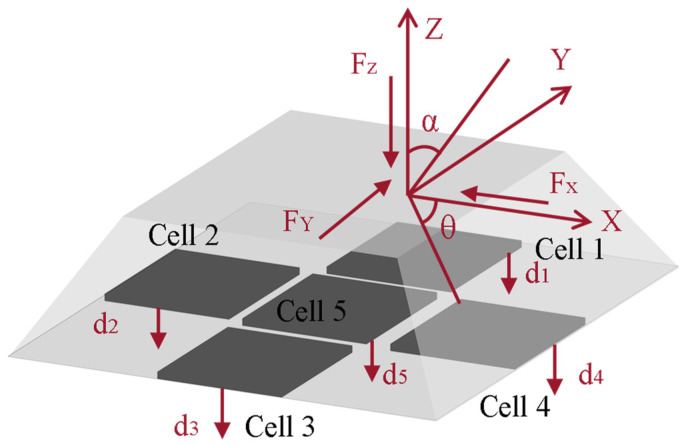
The sensor’s three-dimensional sensing unit and its force sensing.

**Figure 2:**
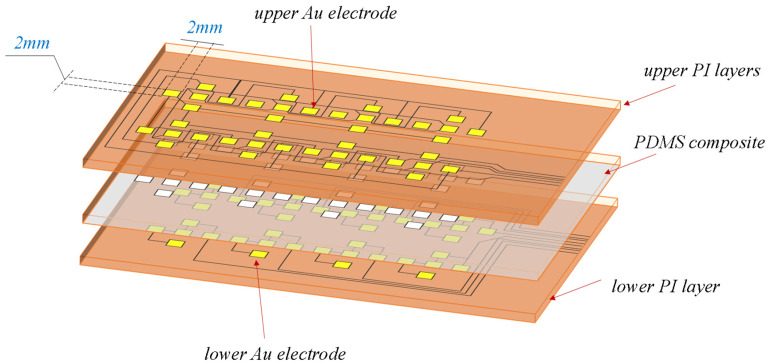
Layout of the tactile units in the sensor.

In this work, we employ a multi-point array capacitive tactile sensor. In our previous research [27,28], flexible tactile sensors with small measurement ranges were designed, which are inadequate to meet the wide measurement-ranging demands of tactile sensors in the context of fall detection for elderly individuals. Hence, the sensors are further extended in their measurement range in this section. The sensing electrodes consist of upper and lower electrode layers with an intermediate capacitive layer. The electrode layers are masked with flexible polyimide (PI) possessing excellent mechanical properties. On the 150 μm-thick polyimide film, a 200 nm-thick copper (Cu) layer is deposited as the upper and lower electrodes. To enhance the bond between the electrodes and the intermediate layer, a thin layer of chromium metal is introduced between them to improve gold adhesion on the intermediate layer. To prevent contact between the upper and lower electrode layers when subjected to external pressure, a PDMS (Polydimethylsiloxane) silicone rubber with remarkable flexibility, elasticity, and thermal performance is employed as a spacer. The intermediate layer, corresponding to the gaps in the upper and lower electrode points, utilizes air as the dielectric. Externally, the sensing electrodes adopt a raised design resembling a square-based to effectively receive three-dimensional contact pressure. Each three-dimensional sensor unit encompasses five capacitive sensing elements distributed around and covered by the raised. These capacitive elements are positioned at the midpoints of each side of the square base and at the center. The force distribution and layout of the sensors are illustrated in Figure 1 and Figure 2, respectively.

**Figure 3:**
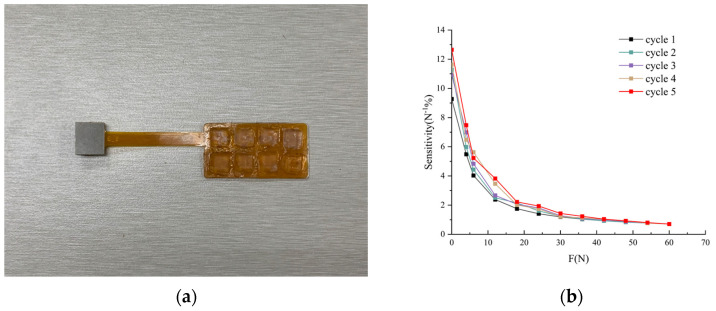
Arrangement of the Tactile Sensor Array and Capacitance Curves: (**a**) Photographic image of the employed array of tactile sensors; (**b**) Capacitance curves of the tactile sensor under applied normal forces.

The overall appearance of the sensor is depicted in Figure 3a. The resistance variation corresponding to the normal force applied downwards is illustrated in Figure 3b, demonstrating its remarkable sensitivity in perceiving both normal and tangential forces. The sensor has a thickness of 1.1 mm, and each three-dimensional force measurement unit covers an area of 7 × 7 mm^2^. By applying normal forces in the range of 0 to 60 N with an accuracy of 2 N along the z-axis to the protrusions on the top of the sensor, the cyclic test results, as shown in Figure 3b, indicate that the capacitance variation maintains a low hysteresis response trend during repetitive measurements. Furthermore, the high sensitivity within the range of 0 to 5 N suggests that the sensor can accurately detect minute contact forces, whereas, within the range of 5 to 60 N, the sensitivity is relatively lower but still capable of measuring larger contact forces. The broad force measurement range makes this sensor suitable for application in scenarios involving human walking.

### 2.2. Analysis of Plantar Tactile Sensor Layout

The distribution of plantar pressure in different regions reflects the human body’s movement state. The primary functions of the human foot are to support the body, cushion and absorb impact forces, facilitate movement, and maintain stability [29,30,31]. As pressure sensors have limited sensing areas, it is crucial to maximize the collection of plantar pressure information. In this study, we aim to analyze the characteristics of plantar pressure and combine them with the features of tactile sensors to determine the optimal layout for the plantar tactile sensor.

Xi et al. [32], based on anatomical considerations, divided the human plantar region into ten areas: first metatarsal region, second-third metatarsal region, fourth-fifth metatarsal region, first metatarsal bone region, second metatarsal bone region, third-fourth metatarsal bone region, fifth metatarsal bone region, medial arch region, lateral arch region, and heel region. During movement, the center of gravity of the body shifts successively from the heel to the metatarsals and toes, while the arch of the foot plays a significant role in cushioning and shock absorption. According to the research by Hallemans et al. [33], when a person is in a static standing position, the heel experiences the highest average peak pressure, followed by the second-third metatarsal region. The regions with lower average peak pressure are mainly distributed in the toe region and the fifth metatarsal region, making the heel, metatarsals, and toes the primary weight-bearing areas in static standing. During walking, the center of gravity gradually shifts forward, leading to increased pressure in the first, second, third-fifth metatarsal regions, and the toe region, while the pressure in the heel region decreases. Based on the above analysis, the key weight-bearing areas of the plantar region are identified as follows: first metatarsal region, second metatarsal region, third-fourth metatarsal region, medial heel region, lateral heel region, and toe region.

For the arrangement of plantar tactile sensors, there are two approaches. The first approach involves covering the entire plantar area with an unrestricted number of sensors to obtain comprehensive plantar information. Although this method avoids the issue of sensor distribution, it can be complex and costly in terms of sensor acquisition and wiring, making it more suitable for fixed installations rather than daily use. On the other hand, the second approach selects optimal placement points at specific positions with fewer sensors to gather plantar information. While this method may result in relatively less diverse tactile data compared to using a full array of sensors, it enables effective data acquisition at a lower cost and without restricting the human body’s motion space. Therefore, we have chosen this method for sensor arrangement.

**Figure 4:**
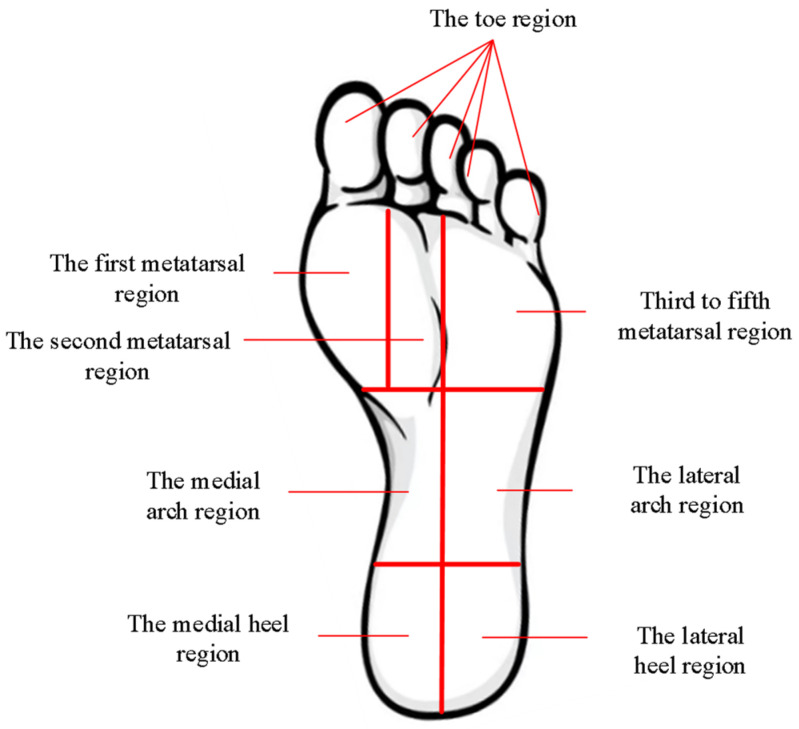
Diagram depicting the division of the plantar regions.

Based on the analysis of the primary weight-bearing areas of the plantar region mentioned earlier, measuring the pressure distribution in the key regions such as the first metatarsal region, metatarsal region, and heel region would be sufficient to obtain the overall plantar pressure distribution. The force during a fall is also closely related to the pressure in these specific areas of the foot. By analyzing and studying the biomechanical characteristics of the plantar region and dividing it based on the key weight-bearing areas within the entire plantar surface, we propose a division into eight regions: toe region, first metatarsal region, second metatarsal region, third-fifth metatarsal region, medial arch, lateral arch, medial heel, and lateral heel. The planar division of the plantar region is illustrated in Figure 4.

During the falling process, when falling forward, the pressure mainly shifts from the heel to the forefoot, transferring to the metatarsal and toe regions. Conversely, when falling backward, the pressure transfer occurs in the opposite direction. The midfoot region essentially forms the arch of the foot, which experiences minimal force due to its arched structure [34]. Therefore, the following regions are proposed as characteristic areas: the toe region, the first metatarsal region, the third-fifth metatarsal region, the medial heel region, and the lateral heel region. When arranging sensors in these characteristic areas, the more sensors are placed, the more accurate the pressure distribution data will be. However, the number of sensors that can be placed on the plantar surface is limited by the sensor’s size, placement method, and connection wiring. Taking practical constraints into account, we select five sensors, each arranged in one of the five characteristic regions.

### 2.3. A Fall Detection Method Based on GCN-LSTM Multi-Task Learning

We define fall detection as a multi-task problem, where the goal is to use temporal tactile data from the plantar region to predict future tactile data and detect walking states (falling forward, backward, left, right, or normal) for multiple time steps ahead. There are two main challenges to address in this context. Firstly, despite using the same tactile sensors and output being positively correlated with force magnitude, it is challenging to extract distinctive feature information from different locations due to their varying sensor layouts. Secondly, the model needs to predict future tactile data based on the input temporal tactile information while simultaneously detecting the corresponding walking states at each time step. To tackle these challenges, we propose a multi-task learning GCN-LSTM model for fall detection. We concretely illustrate the application scenario of fall detection and elaborate on the details of each component in the model.

#### 2.3.1. Problem Formulation

Firstly, considering the layout of the five tactile sensors mentioned earlier, each multi-array sensor consists of eight tactile units, and each unit is represented by a three-dimensional force vector, denoted as $F_{i}=\{f_{i_{x}},f_{i_{y}},f_{i_{z}}\}\in {\mathbb{R}}^{3}$. Additionally, the basic matrix of all tactile sensing units is denoted as $F=\{F_{1},F_{2},\ldots ,F_{40}\}\in {\mathbb{R}}^{40\times 3}$. Since the positions of the tactile sensors are fixed, we construct a static tactile graph, G=(V,E), with fixed edge connections, where V is the set of tactile sensing units, which is expressed as |V|=5×8 vertices(tactile), and E represents the weights of the edge connections between the tactile sensing units, learned from functions E=ϕ(F) and $E\in {\mathbb{R}}^{8\times 8}$.

Regarding the temporal dependency, we set the length of the backtracking time series to T, allowing us to represent the time series vector of tactile units as $X_{i}=\{x_{i}^{1},x_{i}^{2},\ldots ,x_{i}^{T}\}\in {\mathbb{R}}^{T}$. Subsequently, we can use matrix representation $X=\{X_{1},X_{2},\ldots ,X_{40}\}\in {\mathbb{R}}^{40\times T}$ to capture the historical data of tactile units at different time points. For gait classification, where the number of classes is set to 5, we can utilize one-hot encoding to represent the gait classifications as $C=\{C_{1},C_{2},\ldots ,C_{T}\}\in {\mathbb{R}}^{5\times T}$.

The main objective of the model is to predict the gait classification and tactile unit outputs at time T.

The main objective of the model is to predict tactile unit outputs and detect the gait classification at time T. For this purpose, we develop a multi-task learning framework based on GCN-LSTM to extract spatial and temporal features from the tactile unit data, which we define as f. Additionally, the ground truth and predicted values of the tactile unit data are denoted as $y=\{F_{1}^{T+1},F_{2}^{T+1},\ldots ,F_{3}^{T+1}\}$ and $\hat{y}=\{{\hat{F}}_{1}^{T+1},{\hat{F}}_{2}^{T+1},\ldots ,{\hat{F}}_{3}^{T+1}\}$, respectively, while the ground truth and predicted values of the gait classification are represented as $c^{T+1}$ and ${\hat{c}}^{T+1}$. Therefore, the fall detection problem can be viewed as learning the mapping function f under the premises of the static tactile graph G, tactile unit data matrix X, and gait classification C. Based on this, we aim to detect the gait information at time T, as follows:

(1) $$\left[{\hat{y}}^{t+1},\cdots ,{\hat{y}}^{t+T};{\hat{c}}^{t+1},\cdots {\hat{c}}^{t+T}]=f(G;(y_{t-n},\cdots ,y_{t-1},y_{t})\right)$$

where n is the length of the historical time series, and T is the length of the time series that needs to be detected. It is important to note that our multi-task learning model adopts a shared parameter mode, which will be elaborated on in detail in Section 2.3.4.

#### 2.3.2. Graph Convolution Neural Network (GCN)

**Figure 5:**
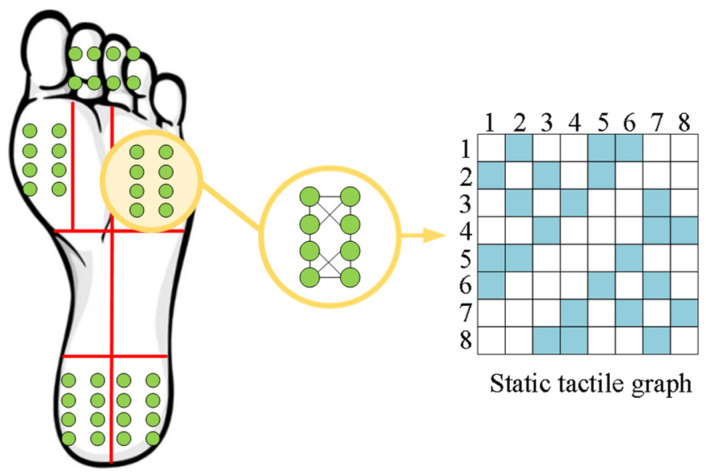
The layout and graph structure of the tactile sensors on the plantar.

Traditional CNNs are suitable for regular two-dimensional grid structures, such as image data, but their applicability is limited when dealing with non-Euclidean structures. Graph Convolutional Networks (GCNs), on the other hand, extend the traditional convolution concept to graph-structured data, enabling the handling of non-Euclidean data and the extraction of relationships between nodes and the topological structure of the graph. Therefore, to address the challenge of extracting feature information from different positions of tactile data due to the varying layout positions of tactile sensors, we employ Graph Convolutional Networks (GCN) to learn the relationships and representations of nodes within the graph structure of the foot sole tactile sensing data. This allows us to capture contextual information between neighboring nodes. We believe that the multi-array tactile sensors on the foot sole exhibit feature connections between adjacent tactile units. By constructing a graph structure, as shown in Figure 5, we can effectively extract information from neighboring tactile units, thereby enhancing the representation capacity of features.

The graph convolutional layer can be represented as a nonlinear function [35]:

(2) $$X^{l+1}=f(X^{l},A)$$

where $X^{0}$ represents the input from the first layer of tactile sensors, $X\in R^{N*D}$, N is the number of nodes in the graph, D is the dimension of each node’s feature vector, and A is the adjacency matrix.

Considering the mutual influence of tactile sensor nodes and addressing the issue of excessive extraction of graph feature information caused by the unnormalized adjacency matrix, our objective is to optimize Equation (2).

(3) $$X^{l+1}=\sigma (D^{-\frac{1}{2}}\hat{A}D^{-\frac{1}{2}}X^{l}W^{l})$$

We introduce the Laplacian matrix $L^{sym}=D^{-\frac{1}{2}}\hat{A}D^{-\frac{1}{2}}$ to address the issue of self-propagation. $\hat{A}=A+I_{N}$ represents the added self-connection matrix, σ denotes the sigmoid activation function, and $W^{l}$ represents the weight of the graph convolution in the first layer. Finally, the extracted features from the graph convolution are fed into the Long Short-Term Memory (LSTM) network discussed in Section 2.3.3.

#### 2.3.3. Long Short-Term Memory Network (LSTM)

**Figure 6:**
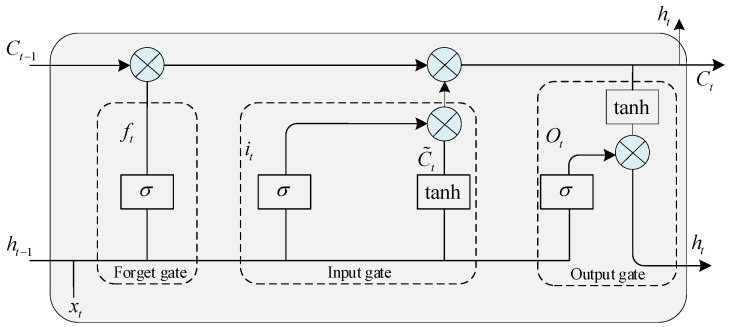
The structure of LSTM.

After extracting spatial geometric features through GCN, we employ LSTM network to further extract the temporal characteristics of the plantar tactile data. LSTM is a specialized type of recurrent neural network that utilizes a cell state to pass and store information while processing sequential data. Among these, the cell state stands as a pivotal component of its architecture, endowed with the capacity to retain crucial information from preceding time steps while judiciously incorporating or discarding data. This imbues LSTM with remarkable efficacy in capturing the intricate web of long-range dependencies inherent in sequential data. It employs three gates (forget gate, input gate, and output gate) to regulate the memory and forgetting of tactile information at each time step. In this approach, the tactile features extracted by GCN serve as input, and LSTM makes decisions based on the input gate to determine which new tactile information will be added to the current cell state. Subsequently, the forget gate controls whether outdated or less significant tactile information is forgotten, ensuring that the cell state retains the most recent and relevant information. Finally, the output gate manages the information within the cell state to produce predictions of the next time step’s tactile data. The basic structure of the LSTM network is depicted in Figure 6.

In contrast to directly using the LSTM method, this study utilizes the feature information extracted by the graph convolutional layer as input. The internal calculations of the LSTM are as follows:

(4) $$f_{t}=\sigma (W_{f}\cdot [h_{t-1},\sigma (D^{-\frac{1}{2}}\hat{A}D^{-\frac{1}{2}}X^{l}W^{l})]+b_{f})$$

(5) $$i_{t}=\sigma (W_{i}\cdot [h_{t-1},\sigma (D^{-\frac{1}{2}}\hat{A}D^{-\frac{1}{2}}X^{l}W^{l})]+b_{i})$$

(6) $${\tilde{C}}_{t}=\tanh(W_{C}\cdot [h_{t-1},\sigma (D^{-\frac{1}{2}}\hat{A}D^{-\frac{1}{2}}X^{l}W^{l})]+b_{C})$$

(7) $$C_{t}=f_{t}*C_{t-1}+i_{t}*{\tilde{C}}_{t}$$

(8) $$o_{t}=\sigma (W_{o}[h_{t-1},\sigma (D^{-\frac{1}{2}}\hat{A}D^{-\frac{1}{2}}X^{l}W^{l})]+b_{o})$$

(9) $$h_{t}=o_{t}*\tanh(C_{t})$$

The terms and symbols involved in the LSTM model are shown in Table 1, where $x_{t}$ represents $D^{-\frac{1}{2}}\hat{A}D^{-\frac{1}{2}}X^{l}W^{l}$.

#### 2.3.4. GCN-LSTM Based on Multi-Task Learning

To address the problem of fall prediction based on sequential tactile data, we divide it into two tasks: predicting future time-step tactile data during walking and predicting the walking state. Both tasks utilize tactile sequential features extracted through GCN and LSTM networks. To enhance the model’s performance and generalization, we employ the Multi-Task Learning Methods Based on Parameter Sharing (MTL) to capture and leverage feature information, enabling fall detection in a multi-task scenario. MTL is a machine learning method where multiple related tasks share the same model parameters. This approach reduces the number of model parameters and utilizes correlated information from multiple tasks to improve the model’s generalization ability.

We define the feature information extracted by GCN and LSTM as:

(10) $$H^{1}=\ldots =H^{M}=H={\left[h_{1},h_{2},\ldots ,h_{N}\right]}^{T}\in R^{N\times d}$$

The output is:

(11) $$y=[y^{1},y^{2}]\in R^{N\times 2},y^{m}={\left[y_{1}^{m},\ldots ,y_{N}^{m}\right]}^{T}\in R^{N}$$

Therefore, the MTL weight update process can be expressed as:

(12) $$(\beta^{*},W^{*},b^{*})=\underset{\begin{array}{l} \beta \in R^{S\times 2}\\ W\in R^{S\times d}\\ b\in R^{S} \end{array}}{\arg\min}\sum_{m=1}^{2}||y^{m}-\sum_{j=1}^{S}g_{j}\beta_{j}^{m}||$$

S represents the number of nodes in the hidden layer, $\beta_{j}^{m}$ denotes the external weight parameter of the $m^{th}$ task in the $j^{th}$ hidden layer node, and m∈(1,2) corresponds to the tasks of gait data prediction and gait detection. For the input ${\tilde{t}}^{m}$ of the $m^{th}$ task, MTL provides the predicted value as follows:

(13) $$f({\tilde{t}}^{m})=\sum_{j=1}^{S}g({\tilde{t}}^{m^{T}}w_{j}^{*}+b_{j}^{*})\beta_{j}^{m*}$$

g symbolizes the sigmoid activation function, $w_{j}={\left[w_{j,1},\ldots ,w_{j,d}\right]}^{T}$, while $b_{j}$ represents the internal weight parameter vector and bias internal weight parameter shared by the backpropagation network.

**Figure 7:**
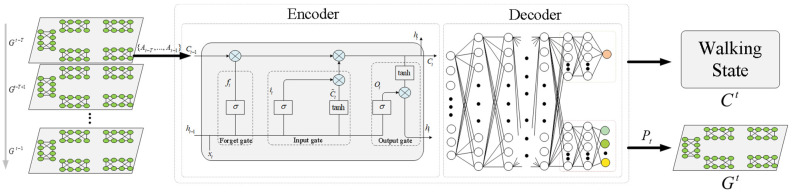
GCN-LSTM Model.

Furthermore, we integrate the GCN, LSTM models, and parameter-sharing multi-task learning method into an end-to-end framework, as depicted in Figure 7. Utilizing GCN, we extract feature information from the past multiple instances of plantar tactile pressure data. Building upon this, the LSTM model extracts temporal features from multiple time steps. Finally, we employ a parameter-sharing multi-task learning approach to capture the spatiotemporal feature information of GCN-LSTM. This enabled us to predict future plantar tactile data ($G^{t}$) and detect walking state ($C^{t}$), thus achieving fall detection during elderly individuals’ walking processes.

## 3. Results

### 3.1. Integrated Nursing Aids with Multiple Flexible Tactile Sensor Arrays

In this work, we introduce a nursing aid device that combines multiple flexible tactile sensor arrays. This device boasts a lightweight design and leverages the flexible and pliable attributes of the tactile sensor arrays. Moreover, the sensors do not require fixation to the ground; instead, they are directly affixed to the soles of the nursing aid, enabling instantaneous and real-time acquisition of tactile data from the feet.

**Figure 8:**
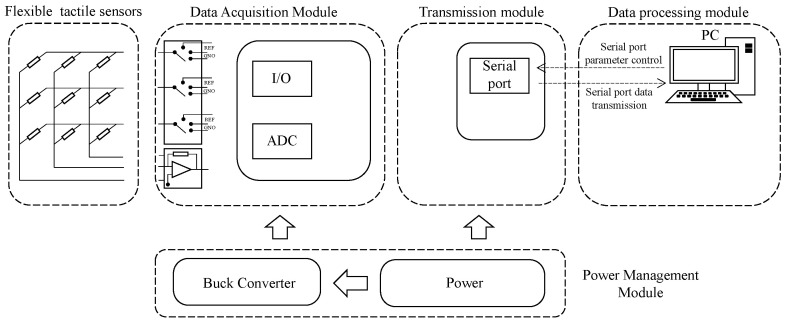
Flexible Tactile sensor system.

**Figure 9:**
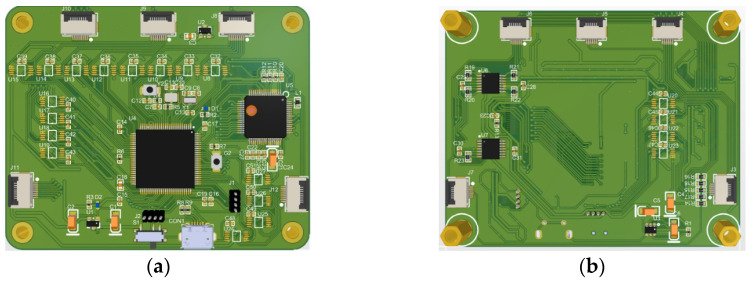
PCB of the electrical circuit system: (**a**) Front view; (**b**) Rear view.

The tactile sensor system, as depicted in Figure 8, consists of tactile sensors equipped with embedded system software for the acquisition and processing of signals. Additionally, it employs serial communication through Bluetooth to transmit the sensor data to the host computer. The hardware components primarily include the array scanning circuit, data acquisition and control circuit, and power management circuit. The array scanning circuit is responsible for detecting voltage variations at each point of the sensor array. To address the issue of inter-array crosstalk current, we effectively employ the zero-potential method [36] for scanning, ensuring efficient crosstalk elimination. The data acquisition and control circuit, on the other hand, governs the operation of the array scanning circuit and the AD acquisition chip. It performs the data collection tasks and communicates the acquired data to the host computer via the serial port. The power management circuit plays a pivotal role in converting the output voltage from the mobile power supply into the required operational voltage, ensuring the stable operation of the circuit. The electrical circuit system, as shown in Figure 9, has dimensions of 7.35 cm×5.9 cm and is equipped with five sets of sensor interfaces. The power management circuit exhibits excellent voltage regulation and stability. It operates with a 5 V direct current power supply, while the circuit requires voltages of 3.3 V, −5 V, and 2.5 V. The 3.3 V voltage sustains the proper functioning of various chips within the circuit, the −5 V serves as a negative voltage reference for the operational amplifier’s inverting amplification circuit, and the role of the 2.5 V voltage is to provide a reference voltage for array scanning.

**Figure 10:**
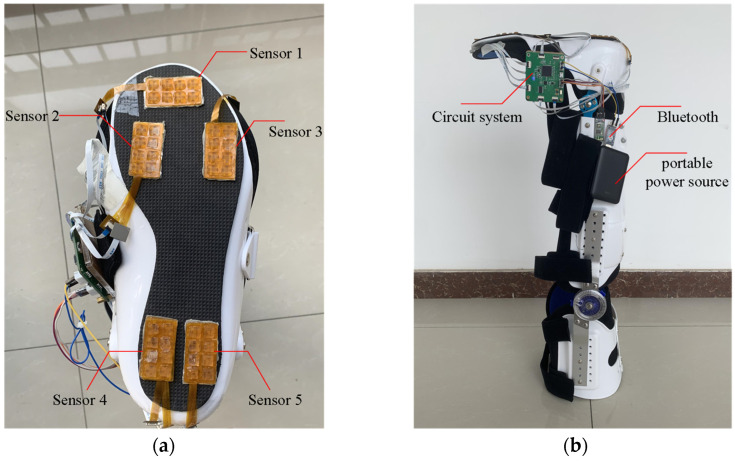
The nursing aid device integrated with multi-flexible tactile sensor arrays: (**a**) Layout and installation of tactile sensors; (**b**) Overall appearance of the nursing aid device.

The tactile sensors can be arranged on insoles, shoe soles, or nursing aids. However, placing pressure sensors on insoles may cause them to shift during movement, resulting in inaccurate pressure readings that do not effectively reflect the force applied. Moreover, this may also impact the lifespan of the sensors. Placing the sensors directly on shoe soles may be affected by the uneven surface, leading to interference with pressure readings. Therefore, in this work, we opt to place the sensors on a relatively flat nursing aid for optimal arrangement and wiring. Building upon the tactile sensor system setup, following the design in Section 2.2, the tactile sensors are installed to the corresponding five characteristic regions of the nursing aid’s sole, as shown in Figure 10a, creating an integrated nursing aid device with multiple flexible tactile sensor arrays. The overall structure of the device is depicted in Figure 10b.

### 3.2. Construction of the Tactile Sequences Dataset for Fall Detection

**Figure 11:**
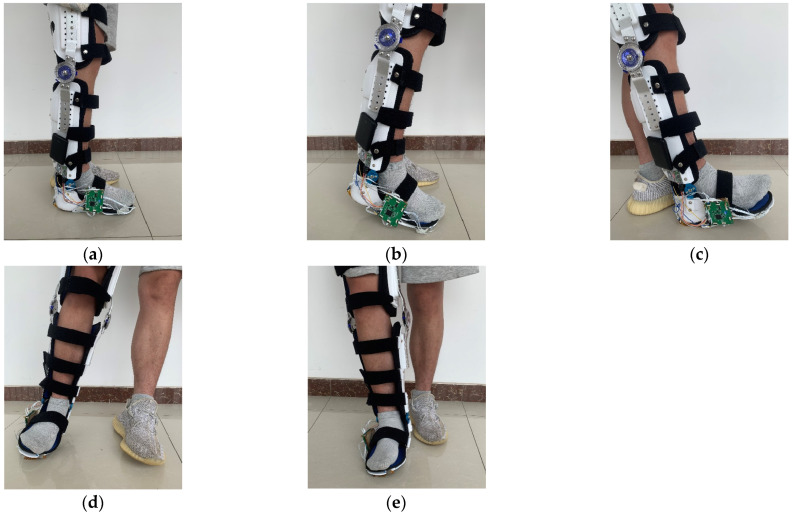
Tactile data acquisition: (**a**) Normal walking state; (**b**) Falling forward state; (**c**) Falling backward state; (**d**) Falling to the left state. (**e**) Falling to the right state.

We recruited five participants to participate in the data collection of plantar tactile information. We used the nursing aid device integrated in Section 3.1 to collect data while the participants walked on tiles under normal walking conditions and simulated falls in four directions: forward, backward, left, and right. At the beginning of each data collection, the participants wire the nursing aid device to their legs and stand in a normal upright position. The data collection process can be divided into the following two parts:

(1) Data collection during normal walking: The participants wire the integrated nursing aid device and walk forward at a speed of 1 m/s, as shown in Figure 11a. The tactile data are collected during the walking process.

(2) Multi-directional falls data collection: While walking normally, the participants are guided to tilt forward, backward, left, and right, as shown in Figure 11b–e. The sensors record the corresponding plantar tactile data and annotate the data with the corresponding fall direction. It is important to note that the continuity of the data collection process should be guaranteed. After collecting data in a single direction, the participants return to normal walking and then tilt in another direction to collect fall data until fall data in four directions are covered.

**Figure 12:**
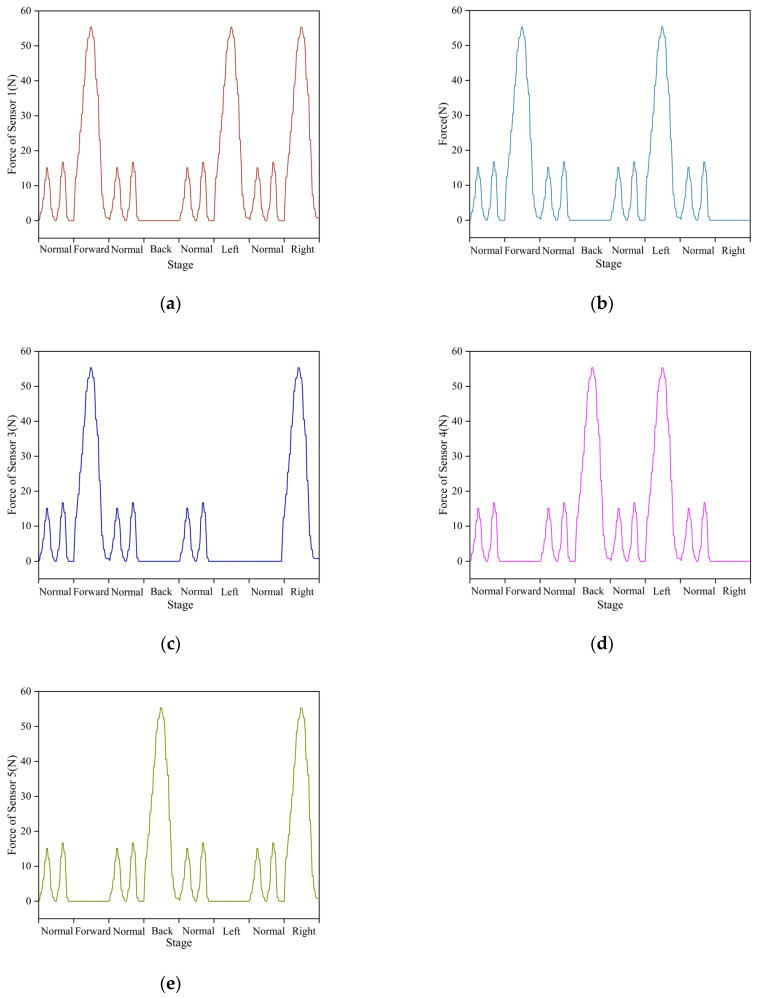
The data from individual tactile units of each tactile sensor: (**a**) Sensor 1; (**b**) Sensor 2; (**c**) Sensor 3; (**d**) Sensor 4; (**e**) Sensor 5. Where “Normal”, “Forward”, “Back”, “Left”, and “Right”, respectively, represent the normal walking state, and the falling states in the forward, backward, left, and right directions.

Each participant underwent 20 tests for each of the five walking states. The tactile sensor collected data at a frequency of 50 Hz, with each collection consisting of 50 frames of tactile time-series data. The tactile dataset consists of approximately 5000 sets of plantar tactile data. We used 4000 sets of plantar tactile data for training the model, and the remaining 1000 sets were used for testing. Partial data are shown in Figure 12, displaying the data collected by sensors 1 to 5. It is observable that sensors at different positions can capture diverse tactile information during different walking states. This collection of tactile information effectively represents the contact perception from different parts during the walking process.

We conduct model training on a platform equipped with a single GPU (NVIDIA GeForce GTX 2080 Ti), a single CPU (Intel i9-10900X 3.7 GHz), and 16 GB of memory. For optimization, we utilize the Adam optimizer with a learning rate of $1\times e^{-8}$ and weight decay of $1\times e^{-6}$.

### 3.3. Model Performance Testing and Comparison

For the prediction of plantar tactile data, we employ widely used metrics [37,38,39] in time series modeling to measure the model’s performance, defined as follows:

(14) $$MSE=\frac{1}{N}{\left(y_{i}-{\hat{y}}_{i}\right)}^{2}$$

where $y_{i}$ represents the true values of plantar tactile data, and ${\hat{y}}_{i}$ represents the predicted values obtained from the model.

To validate the performance of the proposed model, we conduct performance comparisons with several baseline models on the tactile dataset. The results are presented in Table 2.

According to Table 2, our proposed GCN-LSTM model demonstrates superior performance compared to the baseline models. By utilizing GCN and LSTM, it captures the spatial and temporal features of plantar tactile data, enabling the effective prediction of future plantar tactile data at short intervals. Further analysis reveals that the model performs optimally in predicting near-future instances, and its performance gradually decreases over time. We attribute this to the long-term dependencies present in plantar tactile data, which GCN-LSTM might not accurately capture, leading to challenges in predicting data at longer time intervals. However, despite this limitation, GCN-LSTM still outperforms the baseline models and exhibits good overall performance.

For the detection of walking states, we employ *Precision*, *F*1 *Score*, and *Recall* as evaluation metrics:

(15) $$Precision=\frac{TP}{TP+FP}$$

(16) $$Recall=\frac{TP}{TP+FN}$$

(17) $$F1\;Score=2\times \frac{Precision\times Recall}{Precision+Recall}$$

where *TP* represents true positive, *FN* represents false negative, and *FP* represents false positive.

We perform walking state detection using plantar tactile data at different prediction time steps. The results are presented in Table 3.

**Figure 13:**
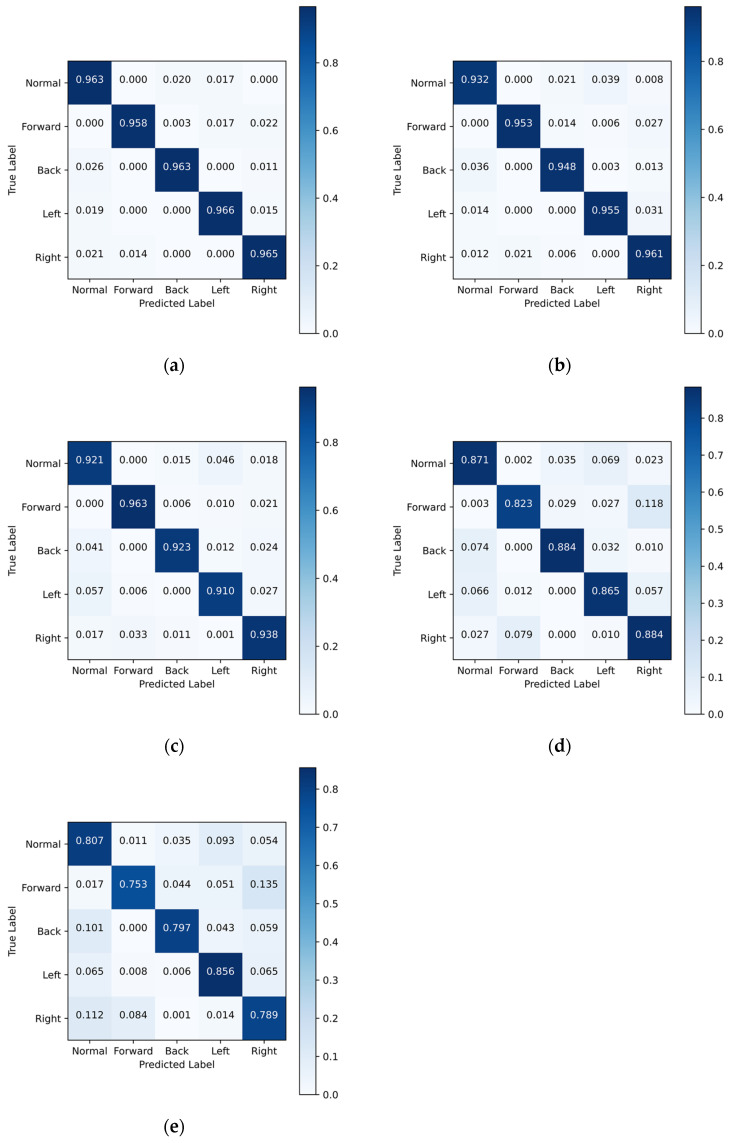
Confusion matrices of the model at detection time steps with 0.2-s interval: (**a**) Time Step 1. (**b**) Time Step 2. (**c**) Time Step 3. (**d**) Time Step 4. (**e**) Time Step 5.

According to Table 3, the model achieves an accuracy of 96.36% at time step 1, demonstrating excellent tactile feature extraction capabilities and effective discrimination of the current walking state. However, as time progresses, the model’s accuracy gradually decreases, reaching 80.03% at time step 5. The confusion matrix is shown in Figure 13. Intuitively, this decline in accuracy can be attributed to reduced confidence in the predicted plantar tactile data, which hinders the model’s ability to extract correct tactile data feature information and leads to a decrease in detection accuracy.

### 3.4. Generalization Testing of Fall Detection on Different Ground Types

**Figure 14:**
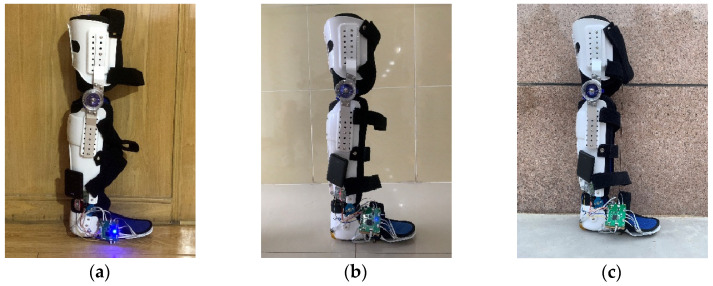
Different ground types: (**a**) Wood Floor. (**b**) Ceramics Floor. (**c**) Cement Floor.

The hardness, roughness, and texture of different ground surfaces bring about differences in tactile data acquired by the tactile sensors. These variations result in different plantar tactile features extracted via the GCN-LSTM model, which can lead to a decline in model performance. Therefore, to test the generalization of the proposed model across different ground types, we conduct tests on three different ground types: wooden floor, cement floor, and ceramics floor, as shown in Figure 14.

**Figure 15:**
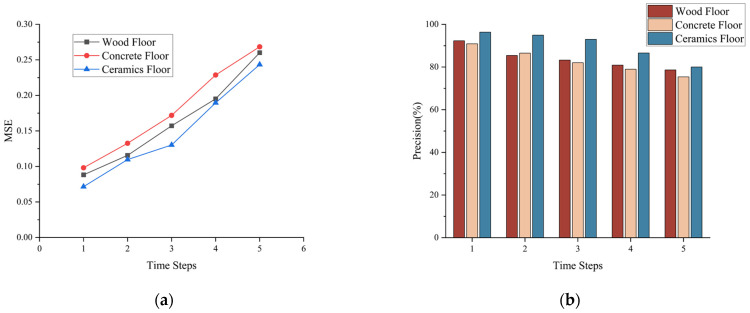
Model performance testing on different ground types: (**a**) Plantar tactile data prediction performance at 0.2-s interval. (**b**) Walking state detection performance at 0.2-s interval.

Figure 15 indicates that compared to ceramic tile, the model’s performance slightly declines on wooden and cement floors. We believe that the data collected by the plantar tactile sensors on different ground types may have inconsistent distributions, and different ground types might introduce varying degrees of noise, making it challenging for the model to fully fit the plantar tactile data. However, our model still demonstrates good performance across different ground types. In terms of plantar tactile data prediction, on the wooden floor, the MSE for time step 1 is 0.0882, and for time step 5 is 0.2601. On the cement floor, the MSE for time step 1 is 0.0981, and for time step 5 is 0.2684. Regarding walking state detection, on the wooden floor, the accuracy for time step 1 is 92.31%, and for time step 5 is 78.65%. On the cement floor, the accuracy for time step 1 is 90.89%, and for time step 5 is 75.41%. This demonstrates that our model can effectively extract plantar tactile data features across different ground types and exhibits good generalization performance.

### 3.5. Generalization Testing of Fall Detection on Different Ground Morphologies

**Figure 16:**
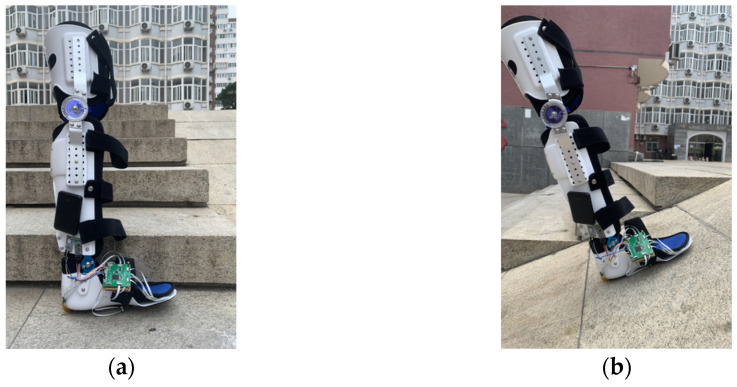
Different ground morphologies: (**a**) Stairs. (**b**) Slope.

Different ground morphologies possess various characteristics, such as height, slope, and texture. These variations in ground morphology may lead to differences in readings from the plantar tactile sensors, thus affecting the model’s performance. Therefore, to test the generalization of the proposed model across different ground morphologies in elderly walking scenarios, we select two ground morphologies: stairs and slopes, as shown in Figure 16.

**Figure 17:**
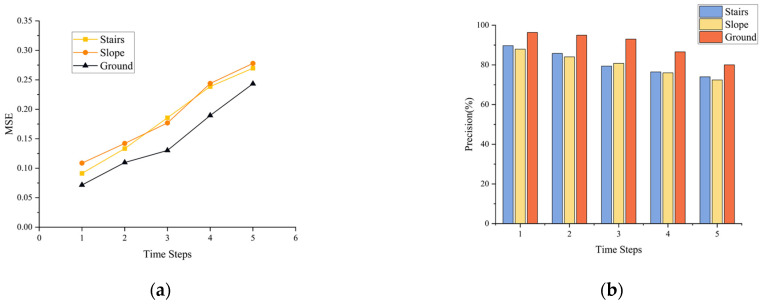
Model performance testing on different ground types. (**a**) Plantar tactile data prediction performance at 0.2-s interval. (**b**) Walking state detection performance at 0.2-s interval.

According to Figure 17, in terms of plantar tactile data prediction, on the stairs, the MSE for time step 1 is 0.0913, and for time step 5 is 0.2698. On the slope, the MSE for time step 1 is 0.1087, and for time step 5 is 0.2779. Regarding walking state detection, on the stairs, the accuracy for time step 1 is 89.71%, and for time step 5 is 74.01%. On the slope, the accuracy for time step 1 is 87.94%, and for time step 5 is 72.38%. The model’s performance on stairs and slopes is lower than on flat ground, suggesting that during the process of climbing stairs or walking on slopes, the plantar tactile data exhibit distribution differences compared to flat ground data. This discrepancy may affect the model’s ability to accurately extract crucial tactile features, resulting in decreased performance. Overall, the model exhibits relatively good generalization performance. It can perform plantar tactile data prediction and walking state detection in different ground morphologies, effectively adapting to the varied walking scenarios in diverse environments.

## 4. Conclusions

In summary, we propose a method for elderly fall detection using nursing aids integrated with multi-array flexible tactile sensors. This method aims to mitigate the risk of falls during the ambulation of elderly individuals. Moreover, it efficiently overcomes the limitations of existing elderly fall detection methods, which often involve higher costs and complex wearable designs. The method leverages tactile sequences collected from the plantar area of the nursing aids during elderly walking to achieve fall detection, demonstrating excellent performance and generalization across various scenarios. We developed a multi-point array capacitive tactile sensor with a convex design using square-based frustum outside the sensing electrodes. This design features five capacitive sensing points symmetrically distributed on the frustum convex, forming a 3D sensor unit with low hysteresis and a wide force range, making it suitable for elderly walking scenarios. Furthermore, we analyzed the plantar force distribution during elderly walking and strategically installed the tactile sensors in the primary force-bearing area of the foot, thereby acquiring relevant and effective tactile data. This approach overcomes the challenges of installing an excessive number of sensors, which would escalate costs, and avoids restricting the body’s motion space caused by fixedly positioning sensors on the ground. On this basis, we introduce a fall detection model based on GCN-LSTM multi-task learning. By utilizing the temporal plantar tactile data, the tactile data and walking states of specific time steps in the future can be detected with high efficiency, which enables real-time gait warnings during the walking process. Five participants were chosen to wear nursing aids for the collection of walking tactile data, resulting in the construction of a plantar tactile dataset. With the plantar tactile dataset, the model achieves an MSE of 0.0716 for predicting the next time step’s plantar tactile data and an accuracy of 96.36% for walking state detection. What is more, the model can achieve fall detection on 5-time steps with 0.2-s intervals in the future and achieve confidence detection results of 80.03% at time step 5. The results outperform other baseline models and demonstrate excellent feature extraction capabilities of the model. What is more, tests on different ground types and ground morphologies are conducted, and the model displays robust generalization and adaptability across these scenarios.

The elderly fall detection model exhibits a reduction in detection success rate over the fifth time step, whose performance is limited in long-time fall prediction. Additionally, the sensors arranged on the soles of the feet experience wear and tear during walking, especially on cement floors, which leads to a decrease in measurement precision and limitations on the long-term application. Besides, due to the recruitment of a small number of participants in our employed dataset, the comprehensiveness of the model could potentially be compromised. In order to improve the efficacy of the method in detecting falls during elderly ambulation, future endeavors will focus on optimizing the long-time prediction performance of the elderly fall detection model, refining manufacturing techniques of the flexible tactile sensors and enhancing the dataset’s scale. Protection measures should also be considered for the sensor design and arrangement guaranteeing long-term reliability.

## Figures and Tables

**Table 1:** The terms and symbols used in LSTM.

Symbol	Definition
σ	Activation function
$x_{t}$	The input feature vector at the current time step
$h_{t-1}$	The hidden state of the previous time step
tanh	Hyperbolic tangent activation function.
$c_{t}$	The cell state at the current time step
$i_{t}$	The output of the input gate
$f_{t}$	The output of the forget gate
$o_{t}$	The output of the output gate
$g_{t}$	Update of the state

**Table 2:** MSE for 0.2-s-interval prediction.

Model	Time Steps				
	1	2	3	4	5
LR [40]	0.2093	0.2431	0.2733	0.3394	0.3725
RNN [41]	0.1348	0.1687	0.2302	0.2677	0.2939
LSTM [42]	0.1203	0.1605	0.2465	0.2581	0.2913
FCNN [43]	0.1687	0.2189	0.2511	0.3347	0.3601
GCN-LSTM(ours)	0.0716	0.1097	0.1302	0.1896	0.2434

**Table 3:** Comparison of Precision, Recall, and F1 Score at detection time steps with 0.2-s-interval.

Time Steps	1	2	3	4	5
Precision	96.36	94.98	93.01	86.56	80.03
Recall	95.12	93.88	92.61	84.97	79.54
F1 Score	95.74	94.43	92.81	85.76	79.78
Time(ms)	2.3	2.3	2.3	2.3	2.3

## Data Availability

The data presented in this study are available on request from the corresponding author. The data are not publicly available due to intellectual property protection.
